# Infodemiological study on the impact of the COVID-19 pandemic on increased headache incidences at the world level

**DOI:** 10.1038/s41598-022-13663-7

**Published:** 2022-06-17

**Authors:** Cristiana Tudor, Robert Sova

**Affiliations:** 1 International Business and Economics Department, The Bucharest University of Economic Studies, 010374 Bucharest, Romania; 2 Management Information Systems Department, The Bucharest University of Economic Studies, 010374 Bucharest, Romania

**Keywords:** Computational biology and bioinformatics, Computational models, Machine learning, Software, Statistical methods, Pain

## Abstract

The analysis of the public interest as reflected by Internet queries has become a highly valuable tool in many fields. The Google Trends platform, providing timely and informative data, has become increasingly popular in health and medical studies. This study explores whether Internet search frequencies for the keyword “headache” have been increasing after the COVID-19 pandemic outbreak, which could signal an increased incidence of the health problem. Weekly search volume data for 5 years spanning February 2017 to February 2022 were sourced from Google Trends. Six statistical and machine-learning methods were implemented on training and testing sets via pre-set automated forecasting algorithms. Holt-Winters has been identified as overperforming in predicting web query trends through several accuracy measures and the DM test for forecasting superiority and has been employed for producing the baseline level in the estimation of excess query level over the first pandemic wave. Findings indicate that the COVID-19 pandemic resulted in an increased global incidence of headache (as proxied by related web queries) in the first 6 months after its outbreak, with an excess occurrence of 4.53% globally. However, the study also concludes that the increasing trend in headache incidence at the world level would have continued in the absence of the pandemic, but it has been accelerated by the pandemic event. Results further show mixed correlations at the country-level between COVID-19 infection rates and population web-search behavior, suggesting that the increased headache incidence is caused by pandemic-related factors (i.e. increased stress and mental health problems), rather than a direct effect of coronavirus infections. Other noteworthy findings entail that in the Philippines, the term "headache" was the most frequently searched term in the period spanning February 2020 to February 2022, indicating that headache occurrences are a significant aspect that defines population health at the country level. High relative interest is also detected in Kenya and South Africa after the pandemic outbreak. Additionally, research findings indicate that the relative interest has decreased in some countries (i.e. US, Canada, and Australia), whereas it has increased in others (i.e. India and Pakistan) after the pandemic outbreak. We conclude that observing Internet search habits can provide timely information for policymakers on collective health trends, as opposed to ex-post statistics, and can furthermore yield valuable information for the pain management drug market key players about aggregate consumer behavior.

## Introduction

The Coronavirus Disease 2019 (COVID-19) pandemic is being dubbed a Black-Swan event ^1^, with significant socio-economic consequences^2–5^, and of high impact on physical and psychological health^6–8^. It is thus widely recognized that COVID-19 has affected health and wellbeing globally, and its impact is multi-faceted^9,10^.

With the pandemic moving into its third year, an accurate assessment of its effects from various perspectives and on different geographies is paramount for estimating the magnitude of the pandemic's impact and most importantly for extracting valuable lessons and informing policy. However, relevant statistics are not published or are published with significant delay, which raises significant challenges for scientific researchers to deliver timely studies^11^.

It would be informative and instructive for policymakers and key players in the global pain management drug market to know whether the pandemic caused an increased occurrence of various pain, although such information is either undetectable, received too late, or both. Suggestions that people are suffering more from headaches in the aftermath of the COVID-19 outbreak have appeared in scientific journals and newspapers^12,13^, Business^14^, Atlantic^15^). Furthermore, quarantine imposed during previous pandemics (i,e. SARS, and Ebola) has been linked to various negative psychological impacts, which could in turn trigger headaches and migraine^16,17^. Consequently, one could reasonably link the COVID-19 pandemic with higher headache incidence. However, an increased occurrence of headache can be attributed both to the direct effect of the virus^18^, or to various collateral pandemic-related factors, including increased concern for personal safety, increased mental health problems, such as anxiety and depression caused by quarantine and lockdown, and resulting from negative socioeconomic outcomes of measures imposed worldwide^4,19,20^.

This assessment is important from at least two perspectives: (1) for policymakers, it provides relevant evidence that helps to accurately assess the impact and consequences of COVID-19; additionally, it informs whether excess occurrences of headache unrelated to direct exposure to the virus could have contributed to straining the health care system over a tumultuous period, thus extracting valuable lessons; (2) for the global pain management drug market players, it provides valuable data on aggregate consumer behavior over distinct geographical regions.

To our knowledge, however, this evaluation has not been accomplished and the topic remains under-investigated, mainly due to the unavailability of official statistics.

To address such challenges, monitoring the health-seeking behavior in the form of public interest reflected by web search queries has emerged as an important tool for early detection of health problems occurrences over certain periods and geographies^21^. Health facilities and insurance firms could potentially report relevant data to highlight incidences of headaches occurring during the pandemic. However, these data are not readily available, and for many countries are never reported. Additionally, even if ultimately reported, these data would only be of service for ex-post research. Furthermore, health facilities provided limited services during the pandemic^22^. Although telemedicine (i.e. the provision of health services to patients in a remote location by using telecommunication technology^23^, and virtual health care emerged as promising alternatives to the classical health-service provision model during the COVID-19 Pandemic^24^ by overcoming the risk of in-person exposure^25^, it is a reasonable assumption that not everyone suffering from headaches sought treatment through health facilities (including by using telehealth) and the insurance system. Thus, given the above considerations, the web search volume queries emerge as the best source of relevant information capable of capturing variations in headache occurrences during the COVID-19 pandemic.

Google has been the absolute leader search engine provider for the past decades, holding a market share of 86.19% as of December 2021^26,27^. Furthermore, the Internet has become an increasingly important source of health information^28^, with over a billion health-related queries passing through Google daily, which accounts for an estimated 7% of Google's daily searches as of 2019^30^. Consequently, Google Trends has been increasingly used over the last decade in the health and medical research^21,31^. Moreover, it is further estimated that the pandemic has increased Internet searches for health information in 2020 compared to 2019 (Statistics Netherlands (CBS)^32^).

Thus, this study sources Google Trends data to extract relevant information for variation in a public health variable (i.e. headache occurrence) at the world level that could not have been otherwise detected. Of note, this study uses “worldwide” web query index data as per the terminology employed by the Google Trends platform, although the term might be misleading due to some noteworthy exceptions (i.e., countries where Google is not active and/or Google Trends data is not available, such as China, the Russian Federation, and most of the African countries). Later in the study, Fig. 3 reflects all countries for which query data is available/unavailable and thus have been included in/excluded from this study. In this respect, the research builds on previous studies that have acknowledged the usefulness of web search data for predicting influenza occurrence, detecting strong correlations between web searches for a health problem, and retrospective surveillance data on that particular health problem reported by official statistics^28,33^; Ginsberg et al., 2009. These studies formed the foundations for the so-called infodemiology (or information epidemiology), a method for studying epidemiology-related data that provides faster access to information compared to standard epidemiological studies and can also uncover otherwise undetectable information^34–38^.

Furthermore, it has been acknowledged that the volume of search queries reflected in Google Trends data is an important predictor of private consumption and retail sales^39–41^. Thus, it is a reasonable assumption that an increase in web searches for a health problem reflects an increase in its occurrence rate that would result in increased private consumption of related medicine.

The main contributions of this paper are threefold:(1) we propose a reliable approach for web query volume forecasting that embeds a pool of relevant statistical and machine-learning forecasting models; (2) we provide a quantitative assessment of excess headache occurrences in the aftermath of the COVID-19 pandemic’s outbreak; and (3) we explore whether the positive link between the pandemic outbreak and increased incidence of headache is a direct consequence of infection or is due to other pandemic-related factors.

Hence, Google Trends query volumes related to the search term “headache” and six statistical and machine-learning predictive models fitted through automated forecasting algorithms are used to estimate headache occurrences in the 6 months following February 2020 and compute excess headache after the pandemic outbreak at the end of January 2020. In this context, COVID-19 excess headaches refer to increases in headaches over what would “normally” have been expected. The “normal” evolution of headache occurrences is projected by the best-perfuming predictive model, which is identified to be Holt-Winters. The best in-sample fit and most importantly, out-of-sample forecasting performance is firstly assured through several accuracy measures and the DM test for forecasting superiority. We find that Holt-Winters is capable of capturing most of the characteristics of the series and producing the most accurate estimations on out-of-same data. Thus, forecasts issued through HW are employed as proxies for the normal evolution of headache web searches. The actual registered values are subsequently compared to the projected “normal” values and excess headache incidence caused by the pandemic is estimated. Results indicate that the pandemic has resulted in excess headaches that would not have occurred in the absence of the pandemic. The countries most affected by headache over the COVID-19 pandemic, as proxied by web searches over the 6 months spanning February 2020–July 2020 are the Philippines, Kenya, and South Africa, whereas before the pandemic most web queries for the keyword were submitted in the Philippines, South Africa, and the US. The study thus concludes that the increasing trend in headache incidence at the world level would have continued in the absence of the pandemic, but that COVID-19 has contributed to its acceleration, causing an excess occurrence in headache (as proxied by web query interest) of 4.53% globally. At the country level, findings show mixed correlations between coronavirus cases and web-searches for the term “headache, which is indicative of a causal relationship running from other pandemic-related factors (i.e. increased stress, anxiety, depression, etc.) to the increased headache incidence. We thus conclude that increased headache occurrence after the COVID-19 outbreak is a result of pandemic-related factors, rather than a consequence of the coronavirus disease.

The remainder of the study is organized as follows. "Previous literature" performs a review of the extant literature. "Data" presents the data employed in the empirical investigation and discusses its historical trends. "Method" illustrates the implemented method. "Results" contains the empirical results and performs robustness checks. "Discussion" discusses the main research findings, and finally, "Conclusion" concludes the study.

## Previous literature

Some aspects that have allowed for timely COVID-19 related research, owing to data availability, are the excess in mortality and hospitalization rates caused by the COVID-19 pandemic, as well as the connection between previous health conditions, such as obesity, and the risk of infection, hospitalization, and death from the novel coronavirus^42^. In this context, COVID-19 excess mortality or hospitalizations refer to mortality/hospitalizations that would not have occurred in the absence of the pandemic^43^. In particular, excess mortality is defined as the surge of all-cause mortality over the level that would have been expected based on historic trends^44^. Consequently, an increasing strand of literature estimates excess death rates^43,45–54^, etc.) or assesses and predicts the spread of COVID-19 as reflected in hospitalization rates^55^ during the ongoing global health crisis. Specifically, previous studies compare actual death rates reported in different countries to those that would have been expected without the black-swan event occurring. This further implies the need for accurate proxies for the level of the variable of interest under “normal” conditions. One approach is to evaluate the excess of deaths compared to the same period in the previous year or an average over recent years (among others ^11,56^). Stang et al.^53^ employ weekly observations and estimate the mean number of deaths corresponding to the 2016–2019 period to estimate expected weekly numbers for 2020. Modig et al.^57^ also use weekly observations for Sweden to identify the baseline level as the average weekly death counts between 2015 and 2019, and subsequently estimate excess mortality by subtracting the baseline level from the observed weekly counts from 2020.

However, this is inconsistent with data that presents strong trends and seasonality. Consequently, another approach has been proposed to correct this inconsistency. This consists in producing estimates for what would have occurred during “normal’ circumstances by using historical data for the variable of interest and employing univariate forecasting models to predict its evolution for the calendar period corresponding to the COVID-19 pandemic. The estimated trend thus proxies for the “normal” evolution of the variable in the absence of the pandemic and is used as a baseline in computing excess (or abnormal) evolutions. Previous studies most often employ the ETS model^58^, the ARIMA model^59,60^, the TBATS model^61^, and the artificial neural network (ANN) models^62^. Some authors estimate multiple models in an attempt to improve inference and forecasting ability. For example, Borrego–Morell et al.^45^ use three statistical methods (i.e. GLM, ETS, and ARIMA) to model the historical evolution and produce estimates of excess mortality rates in Brazil and Spain, and identify ARIMA as overperforming in out-of-sample prediction. The model is then employed to produce estimates for the baseline level with which actual reported rates are compared and excess rates of deaths computed. Perone^55^ estimates ARIMA, ETS, NNAR, TBATS in an attempt to forecast the number of patients hospitalized with mild symptoms and the number of patients hospitalized in the intensive care units (ICU) in Italy, and identifies the best single models as NNAR and ARIMA, while also showing that model combinations generally outperform single models by providing more accurate predictions. Scortichini et al.^63^ estimate a two-stage time-series model, where first a quasi-Poisson time-series regression model with smooth spline functions for time variables and observed predictors is estimated at the province level, and subsequently, the estimates are pooled using a mixed-effects multivariate meta-analysis. Morciano et al.^64^ use data on the number of deaths in all residential and nursing homes in England and apply a Poisson regression model to the pre-pandemic data to forecast the “normal” death count, which is subsequently used as the baseline level in estimating excess deaths. Next, Dahal et al.^65^ first fit Serfling regression models and then employed the generalized logistic growth model to forecast the total excess deaths caused by the COVID-19 pandemic in Mexico. More recently, Talkhi et al.^66^ estimate nine statistical and machine-learning models and conclude that a neural network model (i.e. the multilayer perceptron) and the Holt-Winters model both present the lowest error in forecasting deaths in Iran.

In this study, we implement a similar multi-model approach to explore an outstanding question, specifically the excess headache that has affected the world population over the COVID-19 pandemic period and investigate any potential shift in the population interest before and during the first wave of the COVID-19 pandemic. To produce the baseline level for headache incidence over the testing period, six statistical and machine-learning models (i.e. ETS, HW, ARIMA, STS, TBATS, and NNAR) are estimated through automated forecasting algorithms.

## Data

Google Trends is an online platform that displays the popularity of a search term in a given region over a given period. It offers a time series index of the volume of Google queries submitted in a particular geography. The index reports the query weight or share, expressed as the overall query volume for a particular search term within a geographic region divided by the total number of searches in that region over the period under consideration. Subsequently, the result is scaled on a range of 0–100. Consequently, the maximum query share of a search term for a particular period is normalized to be 100, reflecting the moment at which the search reached the highest popularity. In other words, when the query index is 100, then the ratio of searches for the specific term to the total number of total searches completed in the region is at its greatest point in the specified period. Consequently, the term with the highest interest score is the most frequently searched query within the specified geographical region and period. This is particularly important for extracting the relevancy of web searches in a constantly changing environment.

Consequently, the platform provides a useful tool to measure the popularity of a certain search term among a targeted audience, which can detect health problems early on. In this study, we extract the volume of Google queries for the term “headache” for 5-year period spanning February 15, 2017–February 15, 2022 at the world level. For increased accuracy of results, we have decided toward a smaller, discriminative trend analysis with "terms" as opposed to “topics,” which allows using more targeted, thus more relevant, search queries. It should be mentioned that although headache should be envisioned within a biopsychosocial framework, with biological, psychological, and social/environmental drivers^67^, the search term within the Google Trends platform mainly refers to headache as a symptom associated with physical pain, which is confirmed by the most related queries (as attested subsequently by Fig. 2). This would further imply that people turn to the Internet to search for the term mostly when dealing with the physical symptoms of the health condition.

The final data series contains 261 weekly observations. To investigate potential differences in query trends after the pandemic outbreak, we subsequently split the data into two sub-samples corresponding to the pre-COVID-19 period and the COVID-19 period, respectively. The starting point of the pandemic is set on to be the week spanning January 27th, 2020–February 2nd, 2020, when two main events occurred on the pandemic’s timeline: on January 31st, 2020, the World Health Organization declared the coronavirus outbreak a Public Health Emergency of International Concern and on February 2nd, 2020 the US implemented new travel policies in response to the novel coronavirus disease^68,69^. Consequently, the week of February 3, 2020–February 9, 2020, is the first week in the pandemic subsample or window.

**Figure 1:**
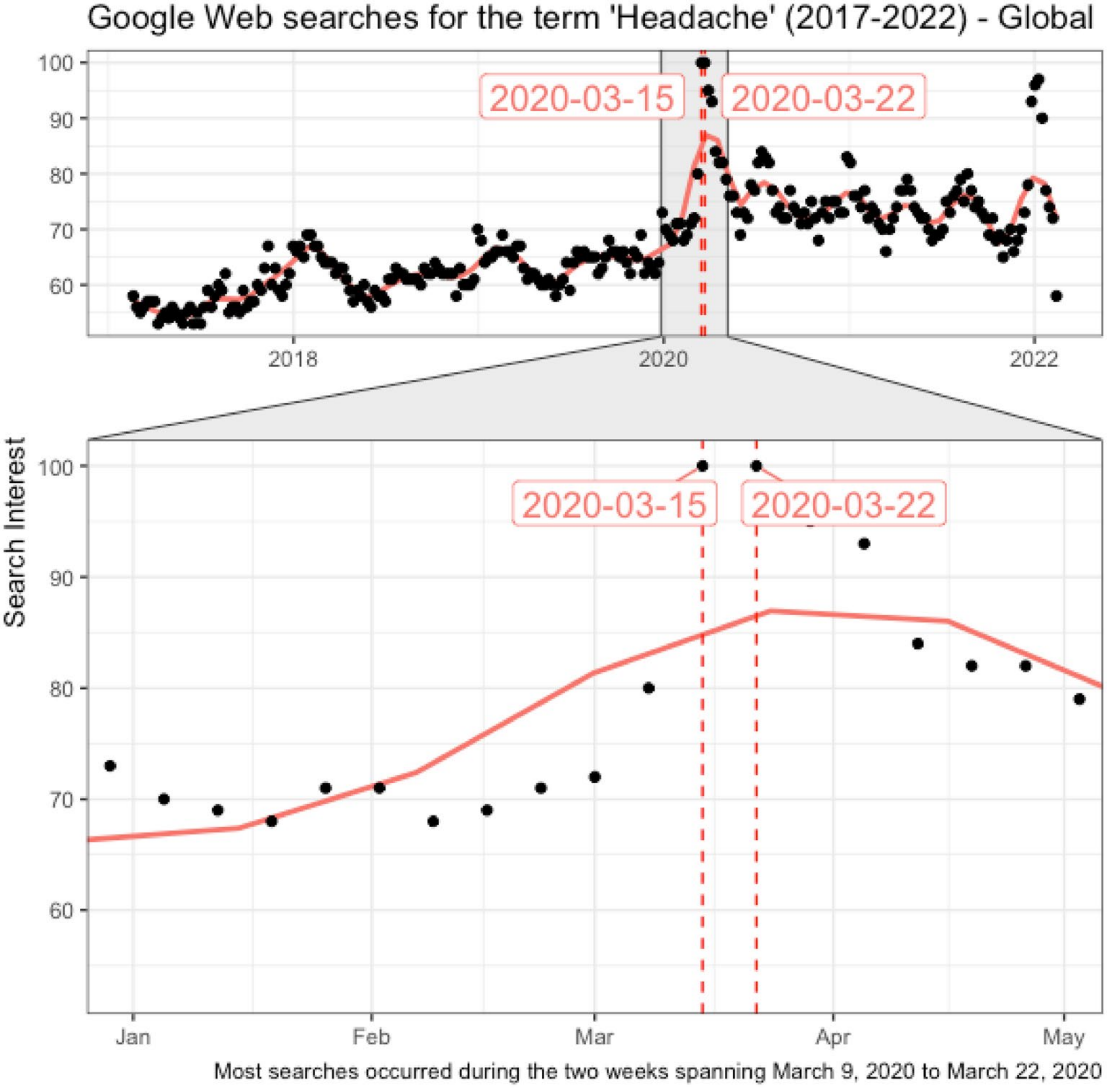
Search volume index for the keyword “headache” (February 2017–February 2022, worldwide) (above); zoom in at the period spanning January to May 2020 (below), reflecting the peak popularity of the term reached on the second and third weeks in March 2020.

Figure 1 reflects that web searches for the keyword “headache” have steadily increased over the last 5 years at the world level. However, the increasing trend has accelerated over the first 3 months of 2020, with the peak popularity of the term reached on the second and third weeks in March 2020 (i.e. the weeks ending on March 15, 2020, and March 22, 2020, respectively). After April 2020, a slightly declining trend in web searches for “headache” emerged, but the overall interest remained higher in comparison to its pre-pandemic levels. The lower part of the chart offers a zoomed view over the first quarter of 2020, highlighting the acceleration of the increasing trend in February 2020 and its peak reached by mid-March 2020.

**Figure 2:**
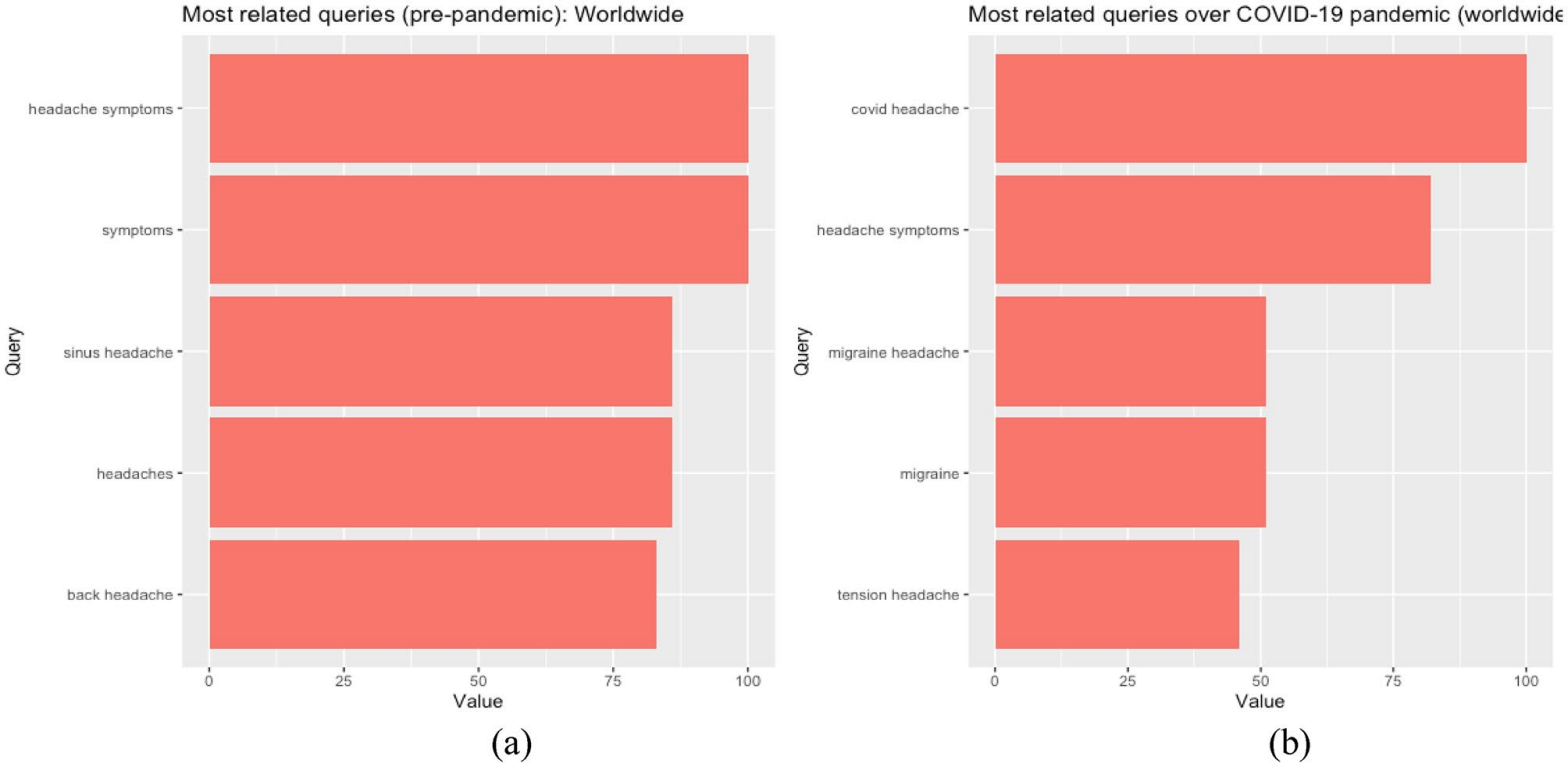
Most related queries to the search term “headache”: Pre-pandemic period (February 2017–January 2020) (**A**); pandemic period (February 2020–February 2022) (**B**).

A deeper look at most related queries in the pre-pandemic and pandemic periods (Fig. 2a,b) indicates a shift from the related term “headache symptoms” before 2020 to “covid headache” during the pandemic period.

**Figure 3:**
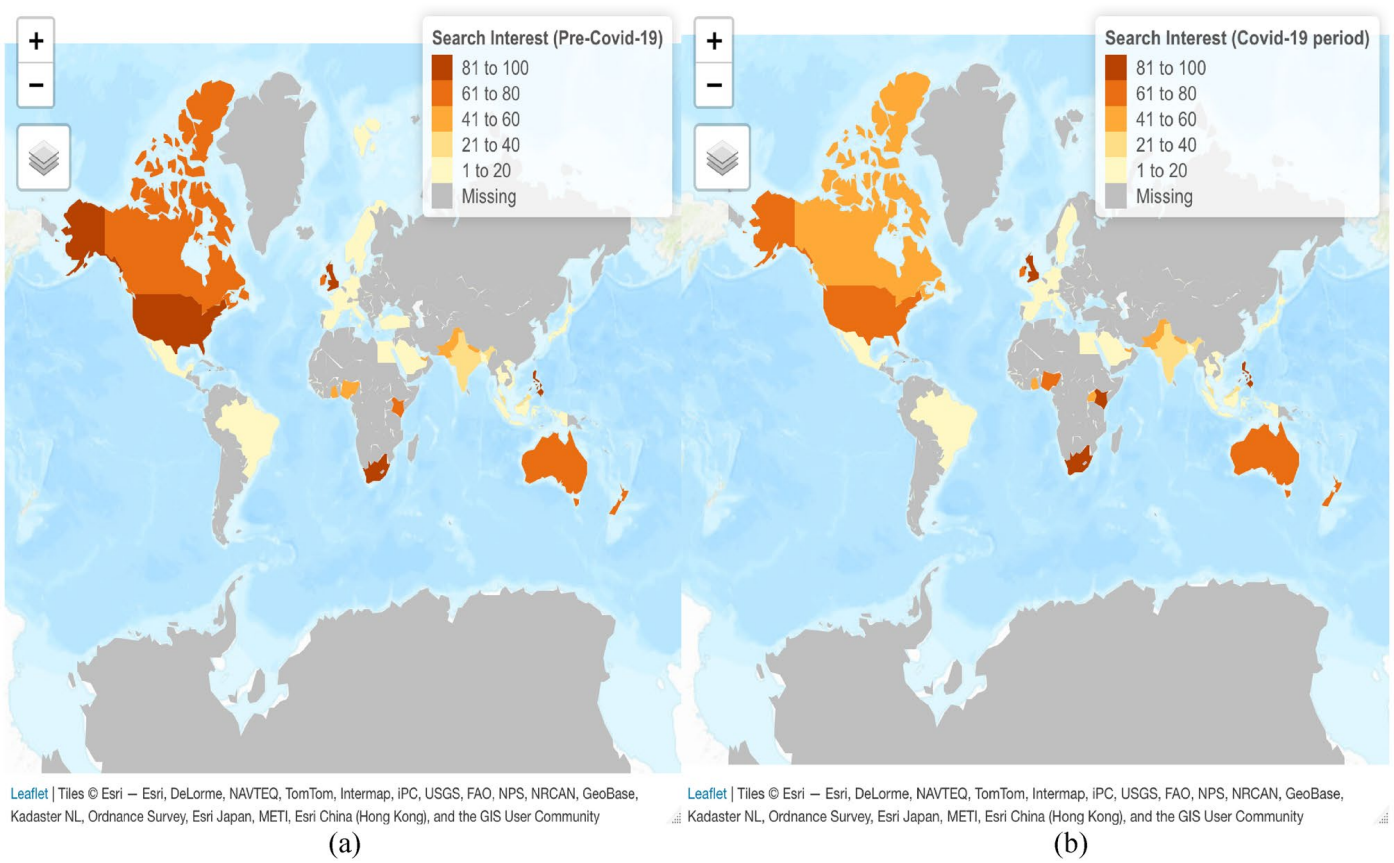
Web search interest (Google Trends) for term “headache” at country level: Pre-pandemic period (February 2017–January 2020) (**A**); pandemic period (February 2020–Febraury 2022) (**B**). Source: Authors’ representation, created with the “tmap” package (https://cran.r-project.org/web/packages/tmap/vignettes/tmap-getstarted.html) in RStudio 2021.9.1.372.

Furthermore, analyzing the search interest for the term “headache” at the country level (Fig. 3a,b), we notice that before the pandemic outbreak the highest query shares are encountered in the Philippines (94), followed by South Africa (92) and the US (89). On the other hand, during the COVID-19 pandemic, the highest query shares are reported in the Philippines (100), Kenya (94), and South Africa (89). There is also heterogeneity in population search behavior at the country level. The relative interest for the key term has decreased after the COVID-19 pandemic outbreak in some countries compared to the pre-pandemic period (i.e. from 89 to 79 in the US, from 69 to 60 in Canada, and from 69 to 64 in Australia), whereas it has increased in others (i.e. from 82 to 86 in the UK, from 32 to 39 in India, and from 42 to 45 in Pakistan).

## Method

To capture most of the characteristics of the time series and thus avoid unreliable forecasts, we employ an array of linear and nonlinear statistical and machine-learning techniques, including Holt-Winters (HW), Exponential Smoothing State Space Model (ETS), Autoregressive integrated moving average (ARIMA), Structural Time Series (STS), Exponential Smoothing State Space Model with Box-Cox Transformation, ARMA Errors, Trend, and Seasonal Components (TBATS), and the neural network autoregression (NNAR). This approach, with the automated implementation of the methods through pre-set automated forecasting algorithms, allows minimizing the risk of model misspecification. Subsequently, the superior model in terms of out-of-sample predictive ability as indicated by several scaled and scale-free model accuracy metrics (i.e. MAE, MAPE, MASE, RMSE, Theil’s U, ACF1) is used to produce forecasts that act as proxies for the evolution of the index of web-queries for the term “headache” during “normal” conditions, or for what could have been expected in the absence of the pandemic.

### Data splitting

To implement the method, the series is split into two subsamples, corresponding to a training period in which the in-sample fit of alternative predictive models is assessed and a testing period (also called a lead-time) in which the best-fit models on the training set are assessed for out-of-sample forecasting ability. We ensure that the two datasets (i.e., the training and testing windows) cover only the pre-pandemic period and, hence, the testing interval does not span into the pandemic timeframe so that any potential shift of data characteristics produced after the outbreak of the COVID-19 pandemic does not affect the out-of-sample accuracy of the alternative predictive models. Moreover, this approach permits to produce forecasts that act as proxies for the evolution of the index of web-queries for the term “headache” during “normal” conditions, or for what could have been expected in the absence of the pandemic. Hence, in the first step, the first 123 weekly observations are employed in-sample for model training and validation, while the data spanning from week 124 to week 155 (i.e., 32 weekly observations ending in January 2020) is used to assess the predictive models' out-of-sample forecasting accuracy. The best forecasting model over this (pre-pandemic) test period is subsequently employed to issue forecasts that cover the first months of the pandemic period spanning February 2020–July 2020 (i.e., a forecasting horizon of 25 weeks). By implementing this forecasting strategy, we thus obtain the most accurate estimates available for the expected volume of web queries for the period spanning February 2020 to July 2020 (i.e., the baseline level) based on weekly historical data from February 2017 to January 2020. Real web-search volume is further compared to forecasted values, resulting in estimations of excess web queries for the term “headache”. The length of the forecasting horizon is carefully chosen to assure, on one hand, that it is smaller than the testing window^70^ and, on the other hand, to mitigate the risk of losing forecasting accuracy that is inherently related to longer forecasts. As per Breitung and Knuppel^71^, longer-term forecasts possibly do not provide any information beyond that contained in the long-run mean of the target variable. Hence, we argue that stretching the forecasting horizon over a longer timeframe would affect the accuracy of the predictive model. Moreover, this approach carries the additional advantage of making the current results directly comparable to previous COVID-19 impact studies.

### Excess query

Excess web-queries are thus computed as the level of unanticipated web searches for the keyword in a week, relative to a “baseline” that represents expected web queries in the absence of the pandemic. Equation (1) depicts the calculation:

1 $$y_{h} = E_{{S_{h} }} - \hat{E}_{{S_{h} }}$$

where $E_{{S_{h} }}$ is the observed level of web-queries index for the term “headache” at time *h* and $\hat{E}_{{S_{h} }}$ is the expected level of web-queries for the respective keyword at *h* under normal conditions. An alternative approach employs averages of past observations over a recent period to stand for the baseline level. However, this method would underestimate expectations for data with trend or seasonality, as in this case, and would thus overestimate excess queries. Consequently, a better approach is to employ a reliable forecasting model to predict the level of web-queries over the period of interest.

### Models

We estimate six widely used models for univariate time series forecasting (ETS, HW, ARIMA, STS, TBATS, and NNAR), identify the best in-sample fit and the best out-of-sample forecasting ability, and subsequently employ the over-performing model to obtain reliable estimates of the expected web queries for 25 weeks spanning from February 2020 to July 2020 based on weekly historical data from February 2017 to January 2020.

R software is employed to implement all methods and carry out estimations.

- The Holt-Winters Forecasting Model (HW), originally presented in Holt^72^ and Winters^73^ contains an exponential component (Et) and a trend component (Tt), such that:
  2 $$E_{t} = wY_{t} + (1 - w)(E_{t - 1} + T_{t - 1} ),0 < w < 1$$
  and
  3 $$T_{t} = v(E_{t} - E_{t - 1} ) + (1 - v)T_{t - 1} ,0 < v < 1$$

Then, the k-step-ahead forecast is given by:

4 $$F_{t + k|t} = E_{t} + kT_{t}$$

HW is automatically estimated through the “HoltWinters” function in the “stats” package in R software^74^. The function performs HW filtering, and automatically finds parameters for the best in-sample HW model by minimizing the squared prediction error.

- Exponential Smoothing State Space Model (ETS) contains three components: the trend (T), seasonal (S), and error (E), and the trend is its turn a combination of a level term (l) and a growth term (b). The trend and seasonal components may be none (N), additive (A), additive damped (Ad), multiplicative (M), or multiplicative damped (Md)^75^. The final model takes the form of a three-character string (Z,Z,Z), where the first letter represents the error assumption of the state-space model, the second stands for the trend type, whereas the third letter identifies the season type^55^. ETS is implemented through the “ets” function in R’s “forecast” package^76,77^, which automatically selects the error, type, and season. Then, the optimal ETS parameters corrected are found via the Akaike information criterion (AICc).
- Autoregressive integrated moving average (ARIMA) models, proposed by Box and Jenkins^78^, are one of the most commonly used statistical methods for linear time series forecasting. A seasonal model ARIMA (p,q,d)(P,Q,D)s is written as in Eq. (5) following the terminology in Hyndman and Athanasopoulos^70^:
  5 $$\begin{aligned} & (1 - \varphi_{1} B - \cdots - \varphi_{p} B^{p} )(1 - \Phi_{1} B^{s} - \cdots - \Phi_{P} B^{sP} )(1 - B)^{d} (1 - B^{s} )^{D} Y_{t} \\ & \quad = (1 - \theta_{1} B - \cdots - \theta_{q} B^{q} )(1 - \Theta_{1} B^{s} - \cdots - \Theta_{P} B^{sQ} )\varepsilon_{t} \\ \end{aligned}$$
  where s is the seasonal period, the lowercase and the capital letters represent nonseasonal and seasonal parameters, and $\varepsilon_{t}$ is a random variable with mean zero and the standard deviation $\sigma$.

ARIMA is estimated through the “auto.arima” function within the “forecast” package in the R software. The function can decide whether the data used to train the model needs a seasonal differencing, and estimates unit root tests, together with the minimization of the AICc and MLE to identify parameters through a step-wise approach.

- Exponential Smoothing State Space Model with Box-Cox Transformation, ARMA Errors, Trend, and Seasonal Components (TBATS) models have been introduced by De Livera et al.^79^. A TBAT model is written as TBATS(ω, p, q, φ, {m1, k1}, {m2, k2}, …,{mT, kT}), where ω is the Box-Cox transformation, k is the number of harmonics used for the seasonal trait, and φ is the dampening parameter. For automated estimation, the function “tbats” from the “forecast” package is used to select optimal model parameters by AIC.
- Structural Time Series (STS) method^80^ asserts a time series is composed of four main stochastic components driven by mutually uncorrelated disturbances^81^.

A generalized expression of the decomposition of a time series is given by:

6 $$y_{t} = \mu_{t} + \psi_{t} + \gamma_{t} + \varepsilon_{t} ,\;\;\;t = 1, \ldots ,T$$

where $\mu_{t}$ is the trend, $\psi$ is the cycle, $\gamma_{t}$ is the seasonal and $\varepsilon_{t}$ is the irregular white noise component. STS is automatically estimated by employing the function “StructTS” in the “stats” package, and finding parameters through maximum likelihood.

**Figure 4:**
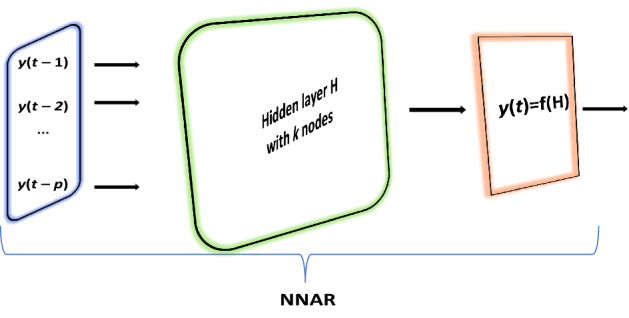
The architecture of a feed-forward neural network autoregression model (NNAR) with *p* lagged inputs, *k* nodes in the hidden layer and the activation function *f*.

- Artificial neural networks (ANNs) are recognized as capable of modeling complex real-world systems owing to their capability to consider non-linearity^82^. An ANN is composed of its basic processing elements (nodes), the connections between nodes, and the activation function^83^. With time-series data, past own values are often used as inputs in an ANN structure, thus developing a neural network autoregression (NNAR) model^84^, as depicted in Fig. 4.

For seasonal data, NNAR models can be described as NNAR (p,P,k)m, where m is the seasonal period, p are nonseasonal lagged inputs for the linear AR process, P are seasonal lags for the AR process, and k represents the number of nodes in the hidden layer. In equation form, the NNAR model reflected in Fig. 4 is given by:

7 $$Y = f(H) = f(W*X + B),\;X = [y(t - 1),\;y(t - 2), \ldots ,y(t - p)]$$

where Y is the output vector of predicted values, f is the activation function, and H = {weight matrix [(p*k)] * input vector} + bias vector (B)^85^.

The NNAR model is fitted through the “nnetar” function within the “forecast” package in R, by making 25 repetitions and minimizing AIC to automatically identify parameters p and P. The number of nodes in the hidden layer is given by k = (p + P + 1)/2.

### Evaluation metrics

Several forecasting accuracy metrics are estimated to explore the forecasting performance of the six models, as follows:

- 8 $${\text{Mean absolute error}}:MAE = \sqrt {\frac{1}{N}\sum\nolimits_{i = 1}^{N} {|y_{i} - \hat{y}_{l} |} }$$
- 9 $${\text{Root mean squared error}}:RMSE = \sqrt {\frac{1}{N}\sum\nolimits_{i = 1}^{N} {(y_{i} - \hat{y}_{l} )^{2} } }$$
- 10 $${\text{Mean absolute percentage error}}:MAPE = mean(|p_{t} |)$$
  where $$p_{t} = \frac{{100e_{t} }}{{y_{t} }}$$
- Mean absolute percentage error:
  11 $${\text{MASE}} = {\text{mean}}(|q_{j} |)$$
  where qj is independent of the scale of the data and is defined as:$q_{j} = \frac{{e_{t} }}{{\frac{1}{N - 1}\sum\nolimits_{i = 2}^{N} | y_{t} - y_{t - 1} |}}$ for non-seasonal series and as: $q_{j} = \frac{{e_{t} }}{{\frac{1}{N - m}\sum\nolimits_{i = m + 1}^{N} | y_{t} - y_{t - m} |}}$ for seasonal time series.
- Theil’s U statistic:
  12 $$Theil^{\prime}s\;U = \frac{{RMSE_{1} }}{{RMSE_{2} }},$$
  where RMSE1 refers to the RMSE metric estimated for the main model and RMSE2 represents the RMSE of the naïve method that always predicts the last observation.
- Lag 1 Autocorrelation of Error:
  13 $$ACF1 = \frac{{\hat{\gamma }(1)}}{{\hat{\gamma }(0)}}$$
  where $\gamma$ is the sample autocovariance function.

For all metrics, the forecast error is given by:

14 $$e_{T + h} = y_{T + h} - \hat{y}_{T + h|T}$$

where {y1,…,yT} is the training window, {yT + 1,yT + 2,…,yN} is the test window, and N the length of the time series.

More details can be retrieved from Hyndman and Koehler^86^ and Hyndman and Athanasopoulos^70^.

### Test for superior predictive ability (SPA)

The test proposed by Diebold and Mariano^87^ and its version further extended by Harvey et al.^88^ has been extensively used in the literature to test the null hypothesis of equal forecast accuracy between two competing models^89^, Diebold^90,91^. The DM test statistic is suitable for forecast errors that are contemporaneously correlated, serially correlated or are non-normally distributed^92^.

The loss associated with forecast i is considered to be a function of the forecast error, eit, which is denoted by g(eit), and which is usually the square or absolute value of eit. Furthermore, the loss differential between the two forecasts is calculated as follows:

15 $$d_{t} = g(e_{1t} ) - g(e_{2t} )$$

and the null hypothesis is:

16 $$HO:E(d_{t} ) = 0,\forall t$$

The DM test is estimated through the “dm.test” function included in R’s “forecast” package.

### Integrated sequential steps for automated forecasting

The following steps are taken to implement the six models in the R environment using automated forecasting algorithms:
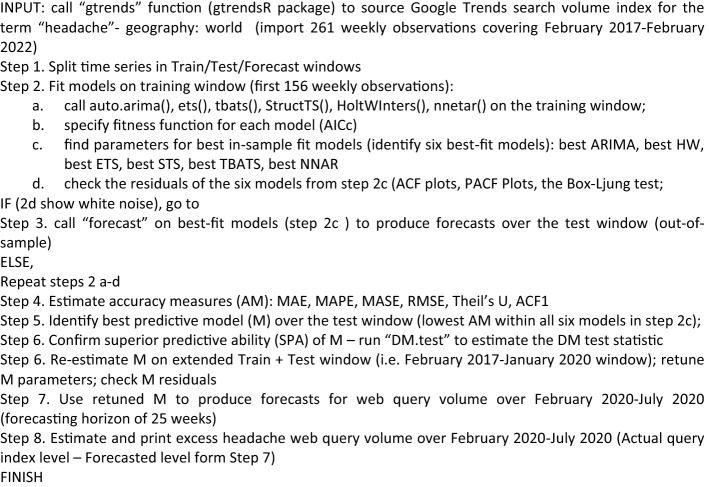


## Results

Table 1 reflects the accuracy measures for the out-of-sample forecasting ability of the six models over the testing period. Results indicate that Holt-Winters and NNAR can provide the best forecast for web-queries, thus over-performing within the universe of six competing models when predicting the level of the web search index over the testing period. Theil’s U statistic is also sub-unitary for the two models, implying that all issued predictions are superior to the naïve method. The other predictive models show significantly higher accuracy measures, indicating that none has been able to satisfactorily capture the evolution of web-queries. Furthermore, the DM test confirms (p value = 0.046) that HW is over-performing within the pool of concurrent models and its forecasting superiority is statistically significant. At the opposite end, ARIMA produces the highest forecast errors for the web-queries series.

**Table 1:** Accuracy measures for the six models.

	ME	RMSE	MAE	MPE	MAPE	MASE	ACF1	Theil's U
mod_hw	0.42	2.64	2.22	0.48	3.37	1.30	0.33	0.88
mod_nnar	0.01	2.91	2.27	-0.18	3.43	1.33	0.43	0.95
mod_sts	2.21	3.61	2.86	3.18	4.25	1.68	0.39	1.20
mod_exp	2.81	4.12	3.24	4.08	4.80	1.90	0.42	1.37
mod_tbats	2.94	4.21	3.33	4.28	4.93	1.95	0.42	1.40
mod_arima	3.16	4.39	3.51	4.62	5.21	2.06	0.41	1.46

Consequently, HW emerges as the optimal choice when a decision should be made toward relying on a single predictive model.

We next employ the HW model to produce the expected values web-queries over the next 6 months corresponding to February-July 2020, when the pandemic started spreading globally. These values are extracted from actual web-search levels produced over the period to arrive at the excess level of queries for the term “headache”. Table 2 centralizes the estimation results.

**Table 2:** Excess headache occurrences (proxied by web-search queries): February 2020–July 2020, 25 weeks.

Week (end date)	Actual query index	Estimated index (HW)	Excess query
09/02/20	68	71	− 3
16/02/20	69	72	− 3
23/02/20	71	72	− 1
01/03/20	72	73	− 1
08/03/20	80	73	7
15/03/20	100	74	26
22/03/20	100	74	26
29/03/20	95	74	21
05/04/20	93	75	18
12/04/20	84	75	9
19/04/20	82	76	6
26/04/20	82	76	6
03/05/20	79	77	2
10/05/20	76	77	− 1
17/05/20	76	77	− 1
24/05/20	73	78	− 5
31/05/20	69	78	− 9
07/06/20	73	79	− 6
14/06/20	72	79	− 7
21/06/20	78	80	− 2
28/06/20	77	80	− 3
05/07/20	82	80	2
12/07/20	84	81	3
19/07/20	83	81	2
26/07/20	82	82	0
	SUM	1913	87
		Excess (%)	4.53

We notice that HW predicted a continuation of the increasing trends in web queries for the term “headache”, indicating that the COVID-19 has accelerated, but not caused, the number of headache occurrences as reflected in web search interest. Estimates indicate an excess level for the web search volume index of 29 over the March 2020–July 2020 period. From March 8, 2020, to April 26, 2020, very high levels of excess web searches for the term "headache" are encountered, but the excess is lost in the following months. This indicates that the pandemic took its main toll on population well-being in the third and fourth months of 2020.

**Figure 5:**
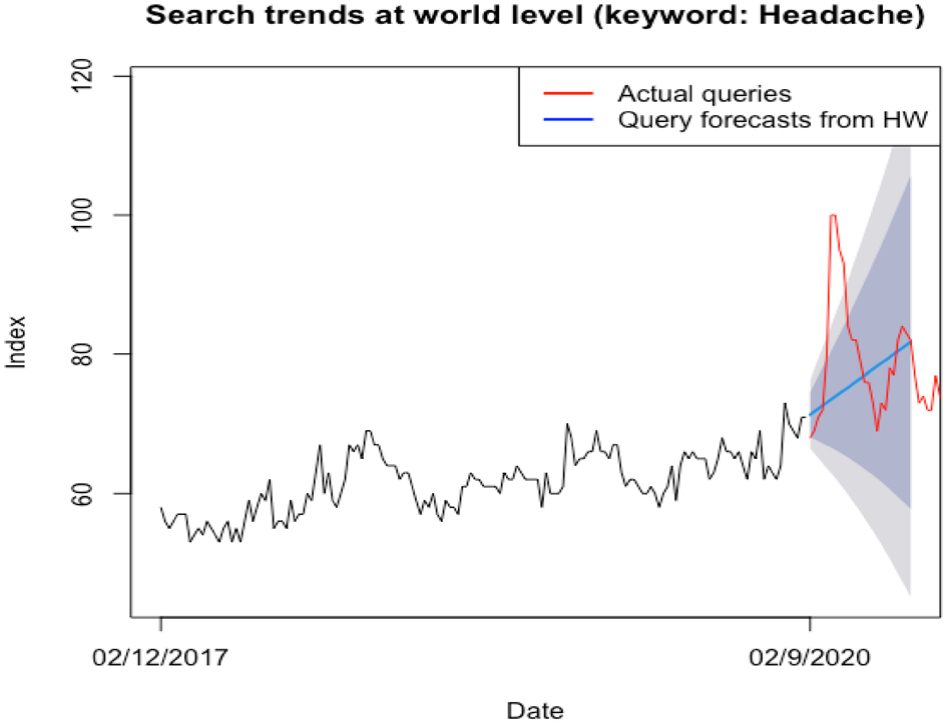
Historical trends and forecasted values for web queries for the term “headache”.

Figure 5 reflects the predictions issued by the HW model for web queries for the term “headache” over the period spanning February 2020 to July 2020 (blue line), along with the real volume number of web searches for the same term, assumed to reflect headache occurrences at world level.

## Discussion

Our research endeavor relies on the works of Polgreen et al.^28^, Ginsberg et al. (2009), Carneiro and Mylonakis^33^, among others, and acknowledges that the Internet is an important source of health information, and web search interest (health-seeking behavior in the form of online queries), is a reliable proxy for a health problem occurrence.

Furthermore, our study is motivated by previous research^34–38^ highlighting that infodemiology can provide faster access to information and is capable to reveal otherwise undetectable information, assisting to improving early detection and formulating effective public health strategies.

Our results indicate, based on Google Trends-query analysis, that headache (as proxied by related web- query volume) would have risen between February 2020 and July 2020 even in the absence of the COVID-19 pandemic. Thus, estimates indicate a “normal” expected growth in headache searches of 15.49% over the 6 months. This prediction is consistent with a rising trend in interest for all pain-related topics, as found by^36^.

However, the actual level of web queries for the term “headache” far exceeded projections (i.e. a 20.59% increase over February 2020–July 2020), indicating that the global health crisis and subsequent measures imposed by governments to contain the spread of the novel coronavirus have affected wellbeing and accelerated headache occurrences worldwide. Our estimations indicate excess headache occurrences of 4.53% relative to expected levels in the absence of the pandemic, or during normal conditions, reinforcing that the COVID-19 has had a significant impact on various aspects of population health. This conforms to Szilagyi et al.^21^, confirming an increased incidence of pain after the outbreak of the COVID-19 pandemic, based on the assumption that Internet searches are a surrogate for the public interest. Findings thus indicate that people are suffering more from headaches in the aftermath of the COVID-19 outbreak, as suggested by previous studies^13^ and media sources (Business^14^, Atlantic, 2021).

However, caution should be exercised when interpreting these results.

On one hand, an increase in the frequency of searches for the term “headache” could in part reflect COVID-19-related symptoms. Headache has been recognized and reported early on as a symptom of coronavirus disease^18^, which could explain the increasing activity on this query.

However, besides the COVID-19 infection, various factors could have also contributed to increased headache incidence over the pandemic period, including anxiety and concern for personal safety, disagreement on optimal treatment, and the lack of viable vaccination options during the first year of the pandemic, the quarantine and lockdown measures imposed to tackle the pandemic, and the negative socioeconomic implications^4,19,20^. Studies that explore the effect of quarantine on population health and wellbeing during previous pandemics^16,17^ bring evidence that supports the latter interpretation.

Consequently, it may seem hard to attribute the increased incidence of headaches to a direct effect of the virus or the other pandemic-related factors. Nonetheless, exploring the shift in the population interest at the country level can shed light on this issue. Hence, mixed correlations are encountered between coronavirus cases and Internet searches for the term “headache. In the US, Canada, Australia, and South Africa, the frequency of web queries decreased after the COVID-19 outbreak, despite the countries reporting high coronavirus infection rates relative to population number. In contrast, in other countries (i.e. UK, India, and Pakistan) the frequency of headache-related searches increased. Hence, the population of the US and UK, two of the most impacted countries worldwide^26,27^ and with similar reported rates of COVID-19 cases (per million population) shows distinct web search behavior. This further supports the argument that the increased web queries are an indication that the increased headache incidence after the COVID-19 outbreak is a result of pandemic-related factors, rather than a consequence of the coronavirus disease.

Moreover, we argue that the results would still have merits in the absence of a clear identification of the driving factors for increased headache incidence. Hence, research findings remain informative for policymakers, assisting in the formulation of specific strategies and for key players in the pain management drug market, providing valuable information for decision-making and business management. The piece that explains the positive link between web searches for a health problem, health problem occurrence, and ultimately private consumption is that the vast majority (i.e. 80%) of Internet users trust the health-related information acquired from web sources^93^.

## Conclusions

The Coronavirus Disease 2019 (COVID-19) pandemic has had a complex, multifaceted, global impact on population health and well-being. However, many aspects of the COVID-19’s impact remain hard or impossible to detect and assess due to the absence or lateness of reported statistics. Hence, there is an increasing strand of literature that estimates excess deaths and hospitalizations during the ongoing global health crisis, although other aspects remain significantly under-investigated. The effect of the pandemic on headache incidence at the world level and in particular countries is one of these outstanding research questions.

In this study, we propose a solution based on assumptions drawn from infodemiology (or information epidemiology), to shed light on the link between the pandemic and headache occurrences at a global level and for specific countries, and to detect any shift in historical trends.

The analysis of Internet-submitted queries has become a highly valuable tool in many fields for early detection of shifts in population interest for specific notions, which in turn could reflect health problems or interest in particular products and services. Google has been the absolute leader search engine provider for the past decades, whereas the Internet has become an increasingly important source of health information for the population worldwide with over a billion health-related queries passing through Google daily as of 2019. Furthermore, there is a recognized strong correlation between web searches for a particular health problem and retrospective surveillance data reported by official statistics, a link that is explained by the acknowledged trust in health-related information acquired from web sources. As a result, the Google Trends platform has become increasingly popular in health and medical studies over the previous decade, providing timely and informative data. Furthermore, the COVID-19 pandemic has increased the volume of Internet searches for health information, further increasing the relevancy and utility of Web-based analysis of search queries.

However, excess headache occurring during the pandemic remains an outstanding research topic, with suggestions indicating increased occurrence, but a quantitative assessment of COVID-19's impact and the investigation of causal relationships are yet to be accomplished.

This study tackles this issue by analyzing the Google search activity of headache-related expressions at the world level and investigating any potential shift in the population interest before and during the first wave of the COVID-19 pandemic. Based on previous findings, this can be indicative of an increased incidence of headache, which in turn has important implications for policymakers and market players, influencing decision-making processes.

Our study thus contributes to extending information epidemiology research and the overall literature concerned with assessing the impact and consequences of COVID-19, by assessing excess headache occurrence after the pandemic outbreak and uncovering causalities through exploring potential shifts in population health-seeking behavior. Moreover, considering the positive link between web queries and ultimate private consumption, identification of the overperforming forecasting method for web-search data contributes to bettering information about future aggregate consumer behavior and improving sales forecasting for the global pain management drug market.

Results indicate that web searches for the keyword “headache” sharply increased globally in the early months of the pandemic, and significantly surpassed the projections based on historic trends. Findings confirm that the COVID-19 pandemic has resulted in an excess occurrence of headaches worldwide that would not have occurred in the absence of the pandemic. Estimations additionally highlight that the increasing trend would have continued in the absence of the pandemic, but COVID-19 has accelerated it. Lastly, research results indicate mixed correlations at the country-level between infection rates and search behavior, suggesting that the increased headache incidence is caused by pandemic-related factors, rather than being a direct effect of the virus.

We argue that Google Trends can be an effective way to analyze trends in public health variables, providing the first indication of health issues even for self-medicated cases that wouldn’t otherwise appear in official statistics. The method employed in this study offers the advantage of generalizability, and can further be employed to explore potential increasing trends in occurrences of other health issues, physical or mental (i.e. diverse pain, depression, anxiety disorders, sleep disorders, suicidal behavior, etc.). Furthermore, the findings are important for extracting valuable information that can assist policymakers in efficiently managing potential future COVID-19 waves or future pandemic events. As such, based on the realistic hypothesis that online searches are a proxy for population interest, detecting an increased search for various medical conditions and/or symptoms after the breakout of a pandemic event would provide relevant and timely information on the symptomatology timeline, as well as on the spread of the condition, that would not otherwise be detected or would be reflected by official health statistics with a significant delay. Moreover, relevant insight into the geographical distribution of increased incidences (as proxied by the web search interest) can inform policymakers and health authorities on the presence of specific health problems of the local population and thus can contribute to the issuance of targeted, more effective policies and strategies.
